# Supplementary material for: Identification and functional analysis of non-coding regulatory small RNA FenSr3 in Bacillus amyloliquefaciens LPB-18
Source: PeerJ. 2023 May 15;11:e15236. doi: 10.7717/peerj.15236 (PMC10194069; doi:10.7717/peerj.15236)
Supplement: Supplemental Information 4 [file peerj-11-15236-s004.zip › KO/CK-vs-T1_map/map04659.html]

KEGG PATHWAY: Th17 cell differentiation - Reference pathway


|  |  |
| --- | --- |
| **Th17 cell differentiation - Reference pathway** |  |

[
Pathway menu
| Organism menu
| Pathway entry
| Show description
| User data mapping
]

Interleukin (IL)-17-producing helper T (Th17) cells serve as a subset of CD4+ T cells involved in epithelial cell- and neutrophil mediated immune responses against extracellular microbes and in the pathogenesis of autoimmune diseases. In vivo, Th17 differentiation requires antigen presentation and co-stimulation, and activation of antigen presenting-cells (APCs) to produce TGF-beta, IL-6, IL-1, IL-23 and IL-21. This initial activation results in the activation and up-regulation of STAT3, ROR(gamma)t and other transcriptional factors in CD4+ T cells, which bind to the promoter regions of the IL-17, IL-21 and IL-22 genes and induce IL-17, IL-21 and IL-22. In contrast, the differentiation of Th17 cells and their IL-17 expression are negatively regulated by IL-2, Th2 cytokine IL-4, IL-27 and Th1 cytokine IFN-gamma through STAT5, STAT6 and STAT1 activation, respectively. Retinoid acid and the combination of IL-2 and TGF-beta upregulate Foxp3, which also downregulates cytokines like IL-17 and IL-21. The inhibition of Th17 differentiation may serve as a protective strategy to 'fine-tune' the expression IL-17 so it does not cause excessive inflammation. Thus, balanced differentiation of Th cells is crucial for immunity and host protection.

|  |  |  |
| --- | --- | --- |
| Reference pathway | 184% 150% 122% 100% 82% 67% 55% | 图片下载 |
